# The Impact of Extraction Orthodontic Treatment on the Impaction of Mandibular Third Molars: An Imaging-based Retrospective Cohort Study

**DOI:** 10.2174/0115734056432921250925055455

**Published:** 2025-10-02

**Authors:** Shuhao Xu, Shiyan Huang, Xiaolong Li, Ping Huang, Wei Li, Xiaoming Zhu

**Affiliations:** 1 Department of Stomatology, Deyang People’s Hospital, Deyang, PR China; 2 Second Clinical Division, Peking University School and Hospital of Stomatology, Beijing, PR China; 3 National Center of Stomatology, National Clinical Research Center for Oral Diseases, National Engineering Laboratory for Digital and Material Technology of Stomatology, Beijing Key Laboratory of Digital Stomatology, Beijing, PR China; 4 Department of Respiratory and Critical Care Medicine, Deyang People’s Hospital, Deyang, PR China

**Keywords:** Midline reference plane, Orthodontic treatment, Orthopantomograms, Premolar extraction, Third molar, Wisdom teeth

## Abstract

**Introduction:**

This study aimed to evaluate the impact of orthodontic extraction treatment on mandibular third molar impaction by measuring changes in angulation before and after treatment in patients receiving extraction *versus* non-extraction orthodontic therapy.

**Methods:**

A retrospective analysis was conducted on 30 patients who completed fixed orthodontic treatment at the Department of Stomatology, Deyang People's Hospital, between 2018 and 2023. Patients were divided into two groups, with 15 included in the extraction group and 15 in the non-extraction group. Pre- and post-treatment orthopantomograms (OPGs) were analyzed, with each mandibular third molar quadrant considered an independent sample. Changes in the α-angle of mandibular third molars were compared between the groups.

**Results:**

The mean change in α-angle was -2.42° ± 8.32° in the non-extraction group and 4.85° ± 9.53° in the extraction group, with a statistically significant difference between the two groups (*p* < 0.05).

**Discussion:**

Whether orthodontic extraction treatment facilitates third molar eruption remains a topic of ongoing debate. Differences in conclusions across studies may be attributed to variations in sample selection, patient age, growth stage, anchorage strategies, and imaging methodology. Our study design attempted to control for these variables by matching participants by age, sex, and treatment duration, and by ensuring comparable baseline α-angle in both groups to minimize confounding. Further prospective studies based on three-dimensional imaging are still needed in the future to validate our conclusions.

**Conclusion:**

Orthodontic treatment involving premolar extraction significantly improved the angulation of mandibular third molars compared to non-extraction treatment, potentially reducing impaction severity and enhancing eruption potential.

## 1 INTRODUCTION

The third molars, commonly referred to as “wisdom teeth”, are the last to develop and erupt in the human dentition. Mandibular third molars begin their intraosseous development between 7 and 21 years of age [1, 2]. With ongoing changes in environmental and dietary factors, human masticatory structures, such as muscles, jaws, and teeth, have undergone asynchronous degeneration [2]. In particular, jawbones have decreased in size more significantly than teeth, leading to a spatial mismatch that often results in third molar impaction due to insufficient eruption space [3]. Consequently, the third molar is the most frequently observed location for impacted teeth, accounting for approximately 98% of all impacted teeth cases, with a reported prevalence ranging from 18.97% to 30.80% [4]. Among these, impaction of the mandibular third molars is the most common clinically encountered subtype [5].

Impacted mandibular third molars can adversely affect oral health by promoting food retention and plaque accumulation, and increasing the risk of pericoronitis, distal caries of the adjacent second molars, periodontal destruction, root resorption, and even cyst formation [6]. Extraction is typically recommended when indicated, but the surgical removal of deeply impacted third molars remains technically challenging. It is often associated with extended operative time, greater bone removal, and elevated risks of nerve injury, bone fracture, emphysema, or root/instrument displacement [7, 8]. Consequently, reducing the severity of impaction, either to avoid extraction altogether or to facilitate a less invasive removal, has become an important goal in clinical practice.

Among the primary contributing factors to third molar impaction are insufficient eruption space and improper angulation [9, 10]. Orthodontic treatment involving premolar extraction has been proposed as a strategy to create space for third molar eruption, potentially improving angulation and reducing extraction difficulty. On the other hand, the association between third molars and anterior dental crowding remains controversial and has been debated since 1969 [11]. Therefore, in recent years, the prophylactic extraction of third molars in adolescents prior to orthodontic treatment has gained increasing popularity, aiming to enhance their long-term stability [12]. Nonetheless, early removal of asymptomatic third molars may pose psychological and neurological risks to young patients [13], whereas delayed removal during or after orthodontic treatment may increase the likelihood of post-operative complications and neurosensory disturbances [14]. Therefore, for patients requiring premolar extraction as part of orthodontic treatment, the timing of third molar removal should be carefully evaluated. To accurately determine the timing of third molar extraction, it is essential to first evaluate whether the orthodontic extraction treatment can meaningfully reduce the severity of third molar impaction. Although numerous previous studies have investigated whether orthodontic extraction treatment can improve the impaction of third molars, their conclusions vary. This discrepancy may be attributed to differences in sample selection, ethnic variations, patient age, growth stages, anchorage strategies, and imaging methods across different studies [4]. Therefore, there is currently no conclusive evidence on whether orthodontic treatment with premolar extraction can improve the impaction status of third molars. This lack of consensus presents a significant dilemma in clinical treatment planning for dentists.

In previous studies, lateral cephalograms, orthopantomograms (OPGs), and cone-beam computed tomography (CBCT) have commonly been used to assess the impaction of third molars. The primary measurement indicators include the eruption space available for the third molars and the angulation of the third molars [10]. Studies have shown that OPGs are a reliable indicator for assessing the position of third molars [15]. Furthermore, since OPGs are routinely performed before and after orthodontic treatment, their use in this study avoided the need for additional examinations of patients. Considering that distance measurements on two-dimensional images may introduce errors, this study utilized the angulation of the third molar (α-angle) as the evaluation criterion for the degree of third molar impaction. Given the higher impaction rates and greater extraction difficulty associated with mandibular third molars, we selected the left and right mandibular third molars as independent analytical units in this study. Our study minimized the influence of confounding factors by matching participants based on age, gender, and treatment duration, while ensuring comparability of the baseline α-angle between the two groups. By assessing pre- and post-treatment changes in mandibular third molar angulation among patients who underwent extraction *versus* non-extraction orthodontic treatment, we aimed to clarify the influence of extraction therapy on mandibular third molar eruption. This may provide a theoretical foundation for pre-treatment decision-making regarding the necessity and timing of third molar extraction to minimize surgical difficulty and related complications.

## 2 MATERIALS AND METHODS

### 2.1 Sample Size Calculation

Based on a previous study the extraction group demonstrated a mean change in α-angle of 8.2° (*SD*=5.4°), while the non-extraction group showed a mean change of 1.3° (*SD*=4.3°) [15]. Using a type I error (*α*) of 0.05 and a type II error (*β*) of 0.2 (power = 80%), the calculated minimum required sample size was 8 participants per group. Given the retro-spective nature of the study and data availability, 15 patients were included in the extraction group. The non-extraction group was matched in a 1:1 ratio based on three stratification factors: gender (male/female), age group (12-18/19-25 years), and treatment duration. In total, 30 patients were included in the final analysis.

### 2.2 Study Design

This retrospective cohort study included 30 patients who completed fixed orthodontic treatment at the Department of Stomatology, Deyang People’s Hospital, between 2018 and 2023. Fifteen patients underwent mandibular premolar extraction (extraction group), and 15 received non-extraction treatment for the mandibular arch (non-extraction group). All treatments were performed by a single experienced ortho-dontist using the same straight-wire technique. Pre- and post-treatment digital orthopantomograms (OPGs) were retrieved from the hospital’s picture archiving and communication system (PACS). All radiographs were acquired under standardized technical settings by a single technician. Each mandibular third molar quadrant was analyzed as an independent sample. Changes in the inclination angle (α-angle) of the mandibular third molars were measured before and after treatment to compare the effects of extraction *versus* non-extraction orthodontic strategies on molar impaction severity.

### 2.3 Participants

The inclusion criteria for the study participants were as follows: (1) age 12-25 years at initial visit and completion of fixed orthodontic treatment; (2) no craniofacial anomalies or congenital tooth absence; (3) pre-treatment OPGs showing bilateral mandibular third molars with >50% crown formation in the pre-eruptive stage; (4) premolar extraction (first or second mandibular premolars) in the extraction group; (5) orthodontic treatment duration of 14-24 months.

Exclusion criteria were as follows: (1) prior orthodontic treatment or history of maxillofacial trauma/surgery; (2) unilateral absence of mandibular third molars; (3) pre-treatment OPGs indicating a strong buccal or lingual eruption tendency of third molars; (4) third molar crowns entirely or predominantly located within the mandibular ramus; (5) fully erupted third molars prior to treatment; (6) orthodontic treatment duration <14 months or >24 months; (7) treatment with removable appliances, clear aligners, or segmental orthodontics.

### 2.4 Study Procedure

Patients’ demographic data, including gender, age at treatment initiation, and treatment time, were collected from the hospital information system (HIS). OPGs were analyzed using Planmeca Romexis software (Planmeca, Finland) to determine landmarks and reference planes. All measurements were conducted by the same orthodontist, who performed landmark identification and angular measurements on both pre- and post-treatment OPGs. Each parameter was measured twice with a two-week interval. If no significant difference was observed between the two measurements (*p* > 0.05), the average was recorded. If a significant difference existed (*p* < 0.05), a third measurement was conducted. The study protocol was approved by the clinical trial ethics committee of Deyang People's Hospital (approval no. 2024-03-039-K01). This study has been registered in the National Medical Research Registry (NMRR) of China (registration ID: MR-51-24-039467).

### 2.5 Measurement of the Impacted Level of Mandibular Third Molars

We selected the angulation of the mandibular third molar to reflect the severity of mandibular third molar impaction. Previous studies have used the occlusal plane and mandibular plane as reference levels to assess the angulation of mandibular third molars. However, both the occlusal plane and mandibular plane may change during growth, potentially leading to errors in the evaluation of mandibular third molar angulation [15]. Therefore, we adopted the α-angle, adapted from a previous study [15], as an indicator of mandibular third molar angulation to assess its impaction severity. The measurement method of the α-angle used for mandibular third molars is described in Fig. (**1**): (1) a vertical line passing through the nasal septum and anterior nasal spine was established as the midline reference plane (MRP); (2) a horizontal reference plane (HRP) perpendicular to the MRP was drawn through the horizontal plate of the palatine bone; (3) the axis of the mandibular third molar (AM3) was defined as the line connecting the mesial contact point of the crown to the midpoint of the furcation area; (4) the α-angle was defined as the angle formed by the intersection of AM3 and HRP.

The change in α-angle was calculated by subtracting the pretreatment value from the posttreatment value. A positive value indicated that the mandibular third molar became more upright, suggesting improved impaction status; a negative value indicated that the tooth became more inclined, suggesting worsened impaction.

### 2.6 Statistical Analysis

All statistical analyses were conducted using SPSS version 22.0 (IBM Corp., USA). The normality of data was assessed using the Kolmogorov–Smirnov test. Data with normal distributions have been expressed as mean ± standard deviation (mean ± SD), while non-normally distributed data have been reported as median (interquartile range). Independent samples t-tests were used for normally distributed variables, and the Mann–Whitney U test was employed for non-normal distributions. A two-tailed *p*-value < 0.05 was considered statistically significant.

## 3 RESULTS

### 3.1 Sample Characteristics

A total of 30 participants (60 mandibular third molars) were included in the study, with 15 patients (30 teeth) assigned to the extraction group and 15 patients (30 teeth) to the non-extraction group. All the baseline characteristics, including age, gender, and treatment time, are summarized in Table **1**. The gender composition was identical between the two groups, with each group consisting of 6 males and 9 females. The average age of the non-extraction treatment group was 13.87±2.75 (range 12-22) years, while that of the extraction treatment group was 14.07±3.03 (range 12-24) years. The average treatment time was 16.33±3.04 (range 14-24) months for the non-extraction treatment group and 17.07±3.47 (range 14-24) months for the extraction treatment group. No statistically significant differences were found between the two groups in terms of the baseline characteristics.

### 3.2 Mandibular Third Molars Angulation

The pre- and post-treatment α-angles of the mandibular third molars are shown in Table **2**. The pretreatment α-angle was 37.45°± 10.93°in the non-extraction treatment group and 35.84°± 9.69°in the extraction treatment group. There were no statistically significant differences in baseline α-angle between the extraction and non-extraction groups (*p*>0.05). The posttreatment α-angle was 35.04°±13.05°in the non-extraction treatment group and 40.7°± 10.32°in the extraction treatment group. The extraction group showed a mean change in α-angle of 4.85° ± 9.53°, while the non-extraction group exhibited a mean change of –2.42° ± 8.32°. The difference between the two groups was statistically significant (*p* < 0.05).

## 4 DISCUSSION

The angulation of mandibular third molars is a critical factor influencing their eruption [9, 10]. Nance *et al*. [16] evaluated the angulation of mandibular third molars relative to the second molar in 237 patients and found that only 11% of teeth with an angulation ≥ 25° and just 3% with ≥35° erupted to the occlusal plane. Vranckx *et al*. [17] further noted that mandibular third molars with an initial angulation > 27.0° tend to worsen in angulation over time.

Currently, several imaging techniques are employed to assess third molar angulation, including lateral cephalograms, OPGs, and CBCT. While lateral cephalograms are routinely obtained in orthodontic patients, they suffer from magnification errors and overlapping structures, which limit their usefulness, particularly when bilateral comparisons are needed [18-20]. CBCT provides precise anatomical visualization, but entails higher radiation doses and cost, making it less suitable for routine use [21, 22]. In contrast, OPGs offer a practical and widely accepted alternative, as they clearly display both mandibular and maxillary molars bilaterally, even in cases of left-right asymmetry [15, 23, 24]. Despite some limitations related to magnification and distortion inherent in two-dimensional imaging, OPGs remain a reliable tool for assessing third molar angulation. Based on these considerations, our study used the α-angle as the primary indicator of impaction severity. Apart from angulation, the available retromolar space also plays a significant role in the eruption potential of third molars [3]. This space is primarily influenced by resorption of the anterior mandibular ramus and mesial drift of posterior teeth during growth [25]. Although the retromolar space is also a significant factor affecting third molar eruption, measurements of eruption space were not conducted in this study due to potential measurement errors associated with OPGs.

Previous studies have confirmed that orthodontic premolar extraction can positively affect the development of the retromolar space [26-28]. However, the routine prophylactic removal of unerupted third molars remains controversial due to potential complications and high costs [29]. Some researchers advocate extraction to reduce long-term crowding or irregularity of anterior teeth [30, 31]. A systematic review covering studies from 1975 to 2015 found that premolar extraction improved both the angulation and eruption space of third molars, while non-extraction treatment had no adverse effect on their development [26]. But several studies argue that premolar extraction does not consistently improve the eruption trajectory of third molars [32]. Table **3** summarizes the findings of recent (2016-2024) clinical studies investigating the impact of orthodontic extraction treatment on third molar impaction. Thus, whether orthodontic extraction treatment improves the impaction status and promotes the eruption of third molars remains a subject of ongoing debate [33-43].

Differences in conclusions across studies may be attributed to variations in sample selection, patient age, growth stage, anchorage strategies, and imaging methodology. Ethnic differences may also contribute to variability in third molar impaction prevalence and characteristics [4]. Additionally, sex-based differences in impaction rates have been reported [10], and these may be attributed to variations in mandibular growth patterns. According to İbişoğlu *et al*. [44], mandibular morphology is significantly associated with third molar impaction. Typically, male mandibular growth continues until around age 20, while in females, jaw growth usually ceases by age 18, prior to the eruption of third molars [45]. Our study design attempted to control for these variables by matching participants by age, sex, and treatment duration, and by ensuring comparable baseline α-angle in both groups to minimize confounding.

Impaction types, such as mesioangular, vertical, horizontal, distal, or inverted, differ in their extraction difficulty. Horizontally impacted molars are generally more challenging to remove and are associated with increased bleeding, tissue trauma, delayed healing, and greater psychological burden [46]. Furthermore, mesial angulation has been strongly linked to root resorption of adjacent second molars [47]. Both difficult extractions and untreated severe impactions compromise alveolar bone integrity and threaten the long-term prognosis of second molars. Therefore, improvement in the angulation of third molars not only contributes to oral health, but also reduces extraction difficulty and minimizes associated surgical complications.

Our findings have indicated the change in the α-angle of mandibular third molars before and after orthodontic treatment to be 4.85°±9.53° in the extraction group and -2.42°±8.32° in the non-extraction group, with a statistically significant difference between the two groups (*p* < 0.05). This suggests that orthodontic treatment with premolar extraction can significantly improve the angulation of third molars, thereby enhancing eruption potential and reducing extraction difficulty. These changes can contribute to better long-term dental health and reduced patient stress.

Nevertheless, this study involved some limitations. First, the anchorage design during treatment was not considered, which may affect molar positioning. Second, skeletal malocclusion types were not evaluated. Finally, the analysis relied on two-dimensional radiographs, limiting our ability to quantify available eruption space. Further prospective studies based on three-dimensional imaging (*e.g*., CBCT) are needed in the future to validate these findings.

## CONCLUSION

This study has demonstrated orthodontic treatment involving premolar extraction to significantly improve the angulation of mandibular third molars compared to non-extraction treatment. These improvements may reduce impaction severity and enhance the likelihood of successful eruption. Strategic space redistribution through extraction appears to facilitate more favorable third molar positioning during development, potentially simplifying future surgical procedures and preserving long-term oral health.

## Figures and Tables

**Fig. (1):**
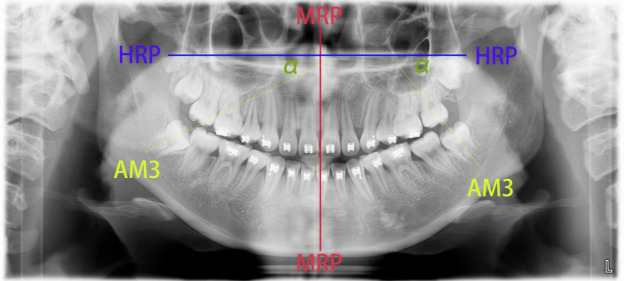
Measurement method of the α-angle for mandibular third molars. (1) A vertical line passing through the nasal septum and anterior nasal spine defined as the midline reference plane (MRP); (2) a horizontal reference plane (HRP) constructed perpendicular to the MRP, passing through the horizontal plate of the palatine bone; (3) the long axis of the mandibular third molar (AM3) defined as the line connecting the mesial contact point of the crown to the midpoint of the furcation area; (4) the α-angle defined as the angle formed by the intersection of AM3 and HRP.

**Table 1:** Demographic baseline characteristics of participants.

-	Non-extraction Treatment Group (*N*=15)	Extraction Treatment Group (*N*=15)	Significance*
Age (years)	-	-	NS
Mean±SD	13.87±2.75	14.07±3.03	
Range	12-22	12-24	
Gender(*N)*	-	-	NS
Male	6	6	
Female	9	9	
Treatment time(month)	-	-	NS
Mean±SD	16.33±3.04	17.07±3.47	
Range	14-24	14-24	

Note: *Independent samples t-test. NS indicates not significant.

**Table 2:** Pre- and post-treatment α-angle of mandibular third molars.

-	Pretreatment	Posttreatment	Differences
Extraction treatment group (*N*=60)	35.84°±9.69°	40.7°±10.32°	4.85°±9.53°
non-extraction treatment group (*N*=60)	37.45°±10.93°	35.04°±13.05°	-2.42°±8.32°
Significance*	-	-	-
*T* Value	0.606	-1.863	-3.148
*p*	0.547	0.068	0.003*

Note: *Independent samples t-test. *p*<0.05 indicates significant.

**Table 3:** Recent studies on the impact of premolar extraction on third molar impaction severity.

Authors\Refs.	Year	Types of Clinical Studies	Grouping and Sample Size	Source of Measurement Data	Measurement Parameters	Results and Conclusions
Peña-Reyes *et al.* [33]	2024	Retrospective Longitudinal Study	-Class I malocclusion with first premolar extractions(n=32) -Class I malocclusion without extractions(n=23) -Class II malocclusion with first premolar extractions(n=24) -Class II malocclusion patients without extractions(n=23)	OPGs	Angulation of mandibular third molar	Class I and II malocclusion extraction treatment exhibited more favorable angulations and a greater number of erupted third molars than non-extraction treatment.
Di Giovanni *et al.* [23]	2024	Cross-sectional study	-Non-extraction (n= 40), -extraction of first lower premolars(n= 40), -Extraction of second lower premolars (n= 40)	OPGs	Angulation and root development of mandibular third molar	Orthodontic treatment involving premolars extractions might be associated with a small acceleration in root development, but not with the angulation, of lower third molars.
Butaya *et al.* [29]	2023	Prospective follow-up	-Premolar extraction(n=36) -Non-extraction(n=98)	OPGs	Angulation, vertical position, relation with the mandibular canal and mineralization status of third molar	Second premolar extractions led to larger retromolar spaces and better third molar angulation in the long term.
Phan *et al.* [24]	2023	Retrospective cohort study	-Non-extraction(n=124) -Extraction of first lower premolars(n=130) extraction of second lower premolars (n= 80)	OPGs	Angulation, vertical position and eruption Space of mandibular third molar	Third molar angulation, vertical position and eruption space significantly improved after orthodontic treatment. The improvement of third molar impaction depended on the extraction indication and orderly reduction in second premolar, first premolar extraction, and non-extracton patients.
Azizi *et al.* [34]	2023	Cross-sectional retrospective study	-First premolar extraction(n = 50) -Non-extraction(n = 50)	OPGs	Angulation of mandibular third molar	Change of mandibular third molar in the extraction group was approximately 3 degrees greater than that in the non-extraction group.
Mang de la ROSA *et al.* [35]	2023	Multicenter retrospective study	-Extraction of premolars (n= 116) -Non-extraction (n=92)	OPGs	Angulation of maxillary third molar	The angulation of maxillary third molars improved over time regardless of the extraction decision.
Janson *et al.* [36]	2022	Retrospective study	-Extractions 2 maxillary and 1 mandibular premolar on the Class I side(n=37) -extraction of 1 maxillary premolar on the Class II side(n=25)	OPGs	Angulations and available space of third molar	Both groups presented better angulation and significantly greater space for third molar eruptions on the extraction quadrants, when compared to the homologous non-extraction quadrants.
Pan *et al.* [37]	2020	Retrospective cohort study	Premolar extraction(n=49)	CBCT	Position, angulation, and rotation of the maxillary third molar	During the process, maxillary third molars moved downward and forward accompanied by outward rotation of the crowns.
Shashidhar *et al.* [38]	2020	Retrospective Study	Extraction of all four first premolars(n=30)	Lateral cephalograms	Angulation of maxillary third molar the mandibular third molar	Maxillary third molars showed more uprighting when compared to the mandibular third molars and that both maxillary and mandibular third molars showed an improvement in their angulations to their respective planes after extraction of the first premolars.
Singh *et al.* [39]	2020	Prospective Study	-First premolar extractions with a high anchorage(n=26) -Non-extraction(n=30)	OPGs	Angulation of mandibular third molar	Extraction therapy in high anchorage cases does not lead to an improvement in third molar angulation.
Azizi *et al.* [40]	2018	Retrospective study	-First premolar extraction with maximum anchorage(n=25) -First premolar extraction with moderate anchorage(n=25) -First premolar extraction with minimum anchorage(n=25) -Non-extraction(n=25)	OPGs	Angulation of mandibular third molar	The first premolar extraction groups with moderate and minimum anchorage may lead to more chance of third molar eruption.
Vitályos *et al.* [41]	2018	Retrospective longitudinal multicentre study	-Premolar extractions(n=53) -Non-extractions(n=174)	OPGs	Position and developmental stage of mandibular third molars	Premolar extraction had a considerably positive influence on the position and development of the lower third molars and therefore could provide better prognosis for their eruption.
Miclotte *et al.* [27]	2017	Retrospective longitudinal study	-Premolar extraction(n=78) -Non-extraction(n=218)	Lateral cephalograms and OPGs	Eruption space, angulation, vertical position, the relation with the mandibular canal and the mineralization status of third molars	Premolar extractions have a positive influence on the eruption space and vertical position of third molars, whereas they do not influence the angular changes of third molars.
Mendoza-García *et al.* [42]	2017	Retrospective longitudinal study	First premolar extraction(n=76)	OPGs	Retromolar space angulation of mandibular third molar	Retromolar space increased significantly post-treatment. The third molar was verticalized in women, while inclination increased among men.
Durgesh *et al.* [43]	2016	Retrospective study	-First premolar extraction(n=90) -Non-extraction(n=90)	OPGs	Angulation of madibular third molar	Extraction of premolars did not demonstrate considerable changes on the angulation of the third molars. The factors other than premolar extractions may influence the angulation of the third molars.

## Data Availability

The authors confirm that the data supporting the findings of this study are available within the manuscript.

## LIST OF ABBREVIATIONS

OPGs: Orthopantomograms
CBCT: Cone-beam computed tomography
PACS: Picture archiving and communication system
HIS: Hospital information system
NMRR: National Medical Research Registry
MRP: Midline reference plane
HRP: Horizontal reference plane
AM3: Axis of the mandibular third molar

## STANDARDS OF REPORTING

STROBE guidelines were followed.
